# Supplementary material for: Effects of a Motor Imagery Task on Functional Brain Network Community Structure in Older Adults: Data from the Brain Networks and Mobility Function (B-NET) Study
Source: Brain Sci. 2021 Jan 17;11(1):118. doi: 10.3390/brainsci11010118 (PMC7830141; doi:10.3390/brainsci11010118)
Supplement: Supplementary file 1 [file brainsci-11-00118-s001.pdf]

Supplemental Materials for:

Effects of a Motor-Imagery Task on Functional Brain Network Community Structure in Older Adults: Data from the Brain Networks and Mobility Function (B-NET) Study

Blake Robert Neyland [1,*], Christina E Hugenschmidt [1], Robert G Lyday [2], Jonathan H Burdette [2], Laura D Baker [1], W Jack Rejeski [4], Michael E Miller [3], Stephen B Kritchevsky [1] and Paul J Laurienti [2]

[1] Sticht Center for Healthy Aging and Alzheimer's Prevention, Department of Internal Medicine Section on Gerontology and Geriatric Medicine, Wake Forest School of Medicine, Winston-Salem, NC 27103, USA; chugensc@wakehealth.edu (C.E.H.); ldbaker@wakehealth.edu (L.D.B.); skritche@wakehealth.edu (S.B.K.)

[2] Department of Radiology, Wake Forest School of Medicine, Winston-Salem, NC 27103, USA; rlyday@wakehealth.edu (R.G.L.); jburdett@wakehealth.edu (J.H.B.); plaurien@wakehealth.edu (P.J.L.)

[3] Department of Biostatistics and Data Science, Wake Forest School of Medicine, Winston-Salem, NC 27103, USA; mmiller@wakehealth.edu

[4] Department of Health and Exercise Science, Wake Forest University, Winston-Salem, NC 27109, USA; rejeski@wfu.edu

* Correspondence: bneyland@wakehealth.edu

Supplemental Methods:

1. The Mobility Assessment Tool – short form (MAT-sf)

The Mobility Assessment Tool – short form (MAT-sf) is a brief measure of older adults' self-perceptions of their ability to perform basic mobility-related tasks such as walking at different speeds and climbing stairs under different constraints. It takes less than 5 minutes to complete and uses 10 video clips of an avatar to depict these tasks that vary in difficulty [1]. The 10 items include: walking on even ground (1), slowly jogging (2), scaling a ramp with (3) and without (4) a handrail, stepping over hurdles (5), walking uphill outdoors on uneven ground (6), going up and down a set of stairs both using (7) and avoiding (8) the use of a handrail, and climbing stairs with one (9) or two (10) bags. Following the viewing of each item, participants are asked about their ability to complete the tasks. For items 1-2, participants are asked the duration for which they could complete the task in minutes between 0 and 60. Questions for items 3 and 4 inquire about the number of times they could complete the task ranging from 0 to 4. The questions following items 5-10 simply ask if participants could complete the task (e.g. yes or no). The 10 items were selected based on individual response and information curves derived from item response theory [1-3]. Information on specific difficulty ratings for each video can be found in the original publication [1].

2. Brain Network Analysis: Study-specific ROIs

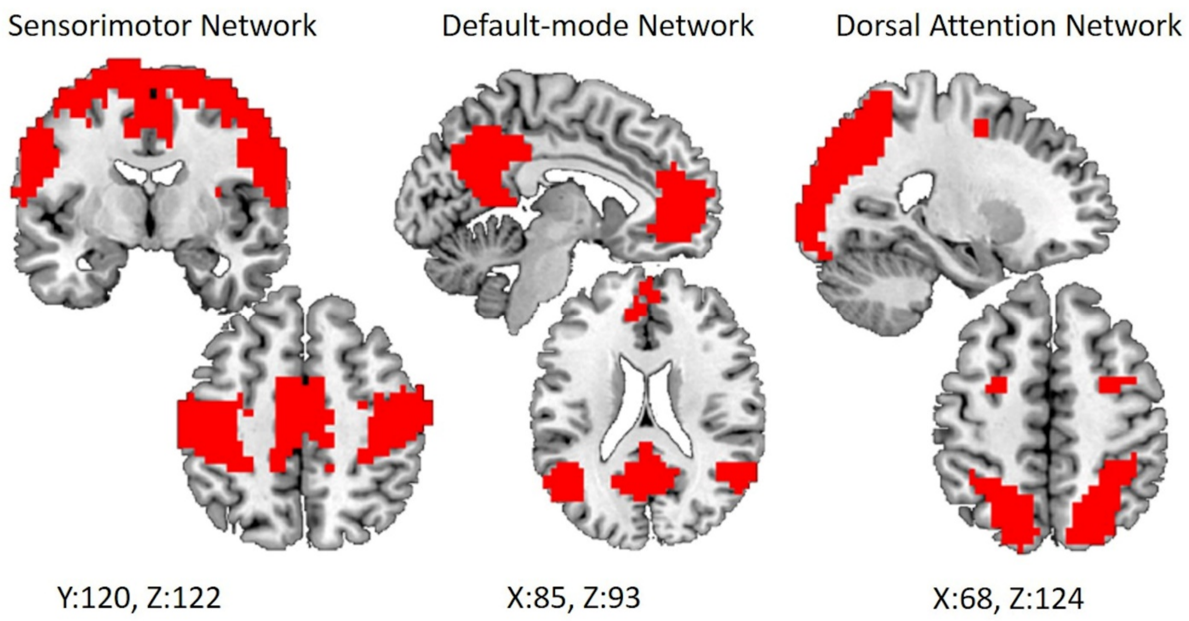

Figure S1: Study-specific regions of interest (ROI) generated for the three intrinsic networks of interest in the regional analyses.

References

1. Rejeski, W. J.; Ip, E. H.; Marsh, A. P.; Barnard, R. T., Development and validation of a video-animated tool for assessing mobility. *The journals of gerontology. Series A, Biological sciences and medical sciences* **2010,** *65* (6), 664-671.

2. Marsh, A. P.; Ip, E. H.; Barnard, R. T.; Wong, Y.-L.; Rejeski, W. J., Using video animation to assess mobility in older adults. *The journals of gerontology. Series A, Biological sciences and medical sciences* **2011,** *66* (2), 217-227.

3. Rejeski, W. J.; Rushing, J.; Guralnik, J. M.; Ip, E. H.; King, A. C.; Manini, T. M.; Marsh, A. P.; McDermott, M. M.; Fielding, R. A.; Newman, A. B.; Tudor-Locke, C.; Gill, T. M.; Group, L. S., The MAT-sf: identifying risk for major mobility disability. *The journals of gerontology. Series A, Biological sciences and medical sciences* **2015,** *70* (5), 641-646.
